# Pol3Base: a resource for decoding the interactome, expression, evolution, epitranscriptome and disease variations of Pol III-transcribed ncRNAs

**DOI:** 10.1093/nar/gkab1033

**Published:** 2021-11-08

**Authors:** Li Cai, Jiajia Xuan, Qiao Lin, Junhao Wang, Shurong Liu, Fangzhou Xie, Lingling Zheng, Bin Li, Lianghu Qu, Jianhua Yang

**Affiliations:** MOE Key Laboratory of Gene Function and Regulation, State Key Laboratory for Biocontrol, School of Life Sciences, The Fifth Affiliated Hospital, Sun Yat-sen University, Guangdong, Guangzhou 510275, P.R. China; MOE Key Laboratory of Gene Function and Regulation, State Key Laboratory for Biocontrol, School of Life Sciences, The Fifth Affiliated Hospital, Sun Yat-sen University, Guangdong, Guangzhou 510275, P.R. China; MOE Key Laboratory of Gene Function and Regulation, State Key Laboratory for Biocontrol, School of Life Sciences, The Fifth Affiliated Hospital, Sun Yat-sen University, Guangdong, Guangzhou 510275, P.R. China; MOE Key Laboratory of Gene Function and Regulation, State Key Laboratory for Biocontrol, School of Life Sciences, The Fifth Affiliated Hospital, Sun Yat-sen University, Guangdong, Guangzhou 510275, P.R. China; MOE Key Laboratory of Gene Function and Regulation, State Key Laboratory for Biocontrol, School of Life Sciences, The Fifth Affiliated Hospital, Sun Yat-sen University, Guangdong, Guangzhou 510275, P.R. China; MOE Key Laboratory of Gene Function and Regulation, State Key Laboratory for Biocontrol, School of Life Sciences, The Fifth Affiliated Hospital, Sun Yat-sen University, Guangdong, Guangzhou 510275, P.R. China; MOE Key Laboratory of Gene Function and Regulation, State Key Laboratory for Biocontrol, School of Life Sciences, The Fifth Affiliated Hospital, Sun Yat-sen University, Guangdong, Guangzhou 510275, P.R. China; MOE Key Laboratory of Gene Function and Regulation, State Key Laboratory for Biocontrol, School of Life Sciences, The Fifth Affiliated Hospital, Sun Yat-sen University, Guangdong, Guangzhou 510275, P.R. China; MOE Key Laboratory of Gene Function and Regulation, State Key Laboratory for Biocontrol, School of Life Sciences, The Fifth Affiliated Hospital, Sun Yat-sen University, Guangdong, Guangzhou 510275, P.R. China; MOE Key Laboratory of Gene Function and Regulation, State Key Laboratory for Biocontrol, School of Life Sciences, The Fifth Affiliated Hospital, Sun Yat-sen University, Guangdong, Guangzhou 510275, P.R. China

## Abstract

RNA polymerase III (Pol III) transcribes hundreds of non-coding RNA genes (ncRNAs), which involve in a variety of cellular processes. However, the expression, functions, regulatory networks and evolution of these Pol III-transcribed ncRNAs are still largely unknown. In this study, we developed a novel resource, Pol3Base (http://rna.sysu.edu.cn/pol3base/), to decode the interactome, expression, evolution, epitranscriptome and disease variations of Pol III-transcribed ncRNAs. The current release of Pol3Base includes thousands of regulatory relationships between ∼79 000 ncRNAs and transcription factors by mining 56 ChIP-seq datasets. By integrating CLIP-seq datasets, we deciphered the interactions of these ncRNAs with >240 RNA binding proteins. Moreover, Pol3Base contains ∼9700 RNA modifications located within thousands of Pol III-transcribed ncRNAs. Importantly, we characterized expression profiles of ncRNAs in >70 tissues and 28 different tumor types. In addition, by comparing these ncRNAs from human and mouse, we revealed about 4000 evolutionary conserved ncRNAs. We also identified ∼11 403 tRNA-derived small RNAs (tsRNAs) in 32 different tumor types. Finally, by analyzing somatic mutation data, we investigated the mutation map of these ncRNAs to help uncover their potential roles in diverse diseases. This resource will help expand our understanding of potential functions and regulatory networks of Pol III-transcribed ncRNAs.

## INTRODUCTION

RNA polymerase III (Pol III) synthesizes a large variety of non-coding RNAs (ncRNAs), such as 5S rRNAs, U6 snRNAs, 7SL RNAs, 7SK RNAs and tRNAs (1–4). These Pol III-transcribed ncRNAs are involved in fundamental cellular processes, such as transcription, RNA processing and protein translation and maturation (1–4). Moreover, these Pol III-transcribed ncRNAs play important roles in various diseases and tumorigenesis (2,3,5). To date, hundreds of Pol III-transcribed ncRNAs have been reported in humans and the mice, and their numbers are likely to grow (4), but the expression, functions, evolution and metabolism of Pol III-transcribed ncRNAs remains largely unclear.

Recent researches have characterized multiple Pol III-associated transcription factors (TFs) to regulate the transcription of Pol III-transcribed ncRNAs (6–10). Importantly, the development of chromatin immunoprecipitation followed by sequencing (ChIP-seq) delineates the genome-wide transcriptional profile of Pol III-associated TFs (11–14). In addition, UV cross-linking and immunoprecipitation coupled to high-throughput sequencing (CLIP-seq) is a technology developed to define the genome-wide profiling of RNA–RBP (RNA-binding protein) interactions (15) and is useful to investigate the functions and mechanisms of Pol III-transcribed ncRNAs. Recently, the transcriptome mapping of RNA modifications gives the possibility to identify RNA modifications on Pol III-transcribed ncRNAs (16,17). Moreover, tremendous amount of small RNA sequencing (sRNA-seq) data generated by multiple consortium projects, such as ENCODE (18), TCGA (19) provides new opportunities to understand the expression and function of Pol III-transcribed ncRNAs. Therefore, it is necessary to integrate these sequencing data to explore the dynamic expression, functions, regulatory network and clinical implications of Pol III-transcribed ncRNAs in physiological and pathological processes.

**Figure 1.**
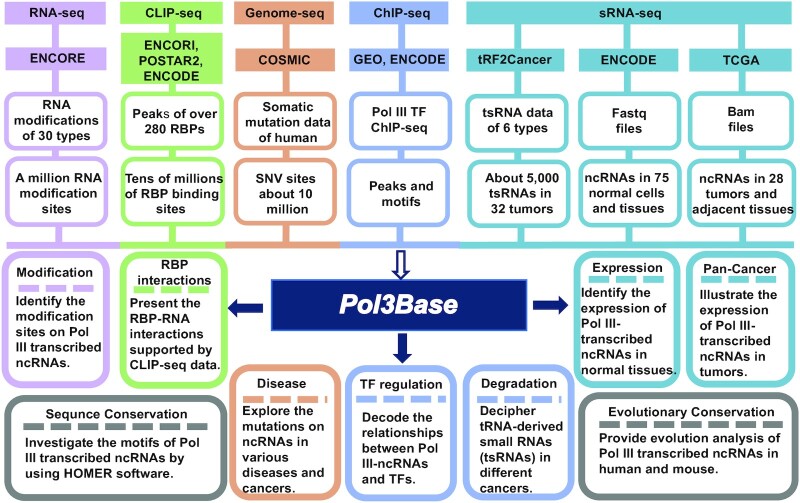
The scheme of Pol3Base workflow. Pol3Base provides the comprehensive integration of Pol III transcriptome of thousands ncRNAs mediated by different TFs. All results generated by Pol3Base are deposited in MySQL relational databases and displayed in web-based pages.

**Figure 2.**
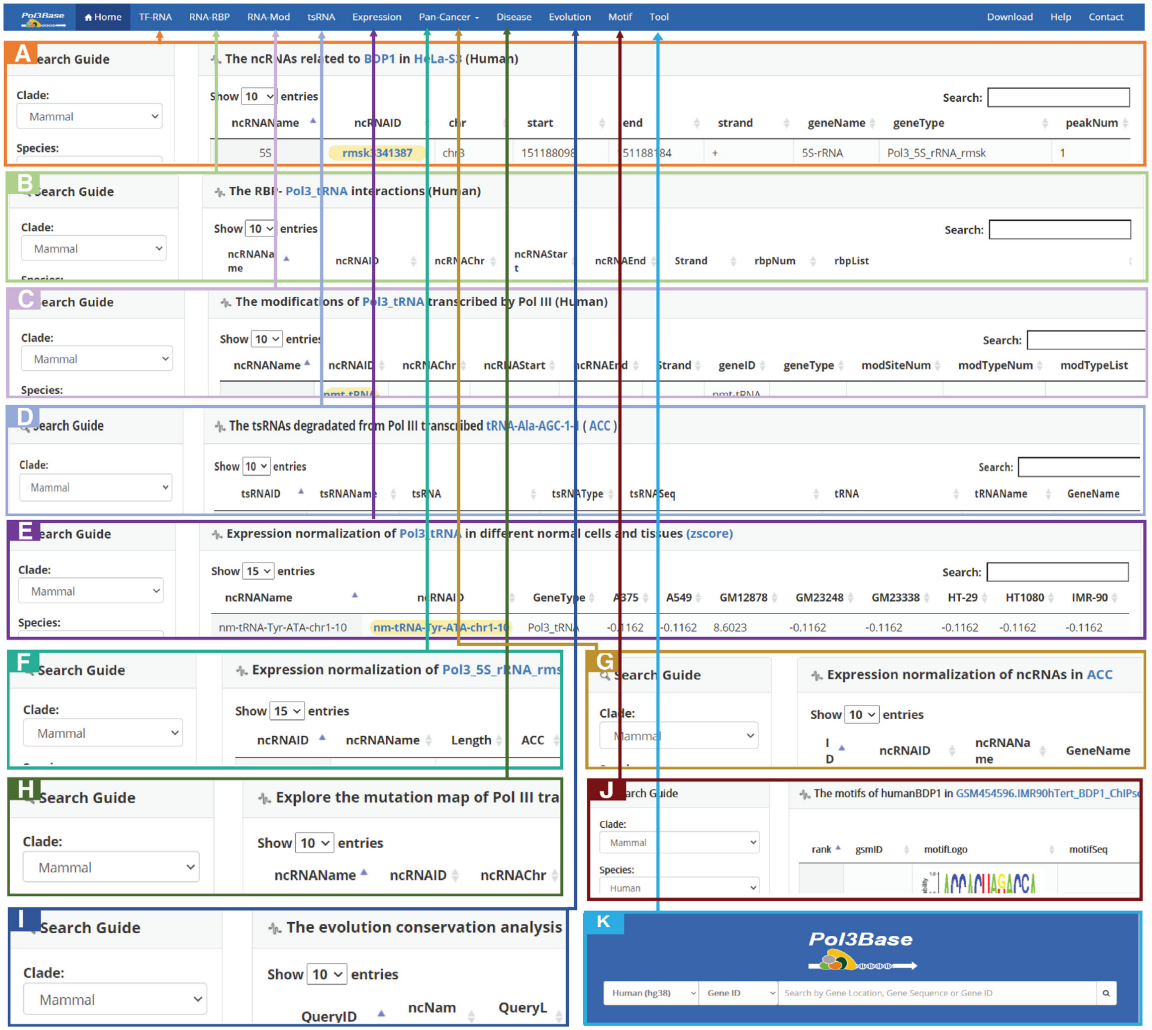
Introduction and usage of Pol3Base. Pol3Base provides multiple web-based interfaces for displaying all Pol III-transcribed ncRNAs interactome, expression, evolution, degradation, epitranscriptome and disease variations. (**A**) Browse ‘TF-RNA’ module for the detailed information of ncRNAs that regulated by 11 TFs. (**B**) The ‘RNA-RBP’ module presents the relationships of Pol III-transcribed ncRNAs with RNA binding proteins (RBPs). (**C**) The ‘RNA-Mod’ module provides the map of RNA modifications on Pol III-transcribed ncRNAs for human and mouse (**D**) The ‘tsRNA’ module explores tRNA-derived small RNAs (tsRNAs) in different cancers. (**E**) The ‘Expression’ module offers the expression profiles of Pol III-transcribed ncRNAs in 75 different cells and tissues. (**F**) In ‘ncRNA case’ of ‘Pan-Cancer’ module, users can select targeted ncRNA type and normalized method to see their expression profiles in different tumors and their adjacent tissues. (**G**) In ‘cancer case’ of ‘Pan-Cancer’ module, users can select targeted tumor type to see the expression levels of all ncRNAs in this tumor and their adjacent tissues. (**H**) The ‘Disease’ module explores the five types of mutation on Pol III-transcribed ncRNAs in 29 different diseases and cancer. (**I**) Evolutionary conservation of ncRNAs in human and mouse in the ‘Evolution’ module. (**J**) The ‘Motif’ module provides the conserved consensus sequence in ncRNAs transcribed by Pol III complex. (**K**) The ‘Tool’ module provides three functions for gene location, gene sequence or gene ID searching.

In this study, we developed Pol3Base (http://rna.sysu.edu.cn/pol3base/ or http://biomed.nscc-gz.cn/DB/Pol3Base/) for decoding the interactome, expression, evolution, epitranscriptome and disease variations of Pol III-transcribed ncRNAs from multi-omics sequencing data (Figure 1). In Pol3Base, we performed a large-scale integration of Pol III-transcribed RNAs in human and mouse, and deciphered their regulatory relationships with dozen of TFs in various cells. Pol3Base also illustrated the global map of RNA modifications in Pol III-derived ncRNAs. Combining with CLIP-seq and sRNA-seq, we investigated the association between Pol III-derived ncRNAs and >200 RBPs and detected tRNA-derived small RNAs (tsRNAs) in 32 different tumor types. Importantly, we elucidated the expression profiles of these ncRNAs in 75 normal tissues and 28 cancer types and their adjacent tissues. We further characterized the mutation map on Pol III-transcribed ncRNAs in 29 different diseases and cancers by analysis of somatic mutation data. Notably, we probed the common features and evolutionary conservation of these ncRNAs between human and mouse, which may provide a valuable lesson for studying their functions in different species. Pol3Base provides a variety of web modules and graphic visualizations to investigate the potential functions and mechanisms of Pol III-derived ncRNAs (Figure 2).

## MATERIALS AND METHODS

### Integration of public ChIP-seq datasets

Human and mouse ChIP-seq datasets for the well-known TFs in RNA polymerase III complex (20), including POLR3D, POLR3GL, POLR3G, BRF1, TFIIIC-220, TBP, BRF2, BDP1, TFIIIC-110, TFIIIC63 and RPC155, were retrieved from the Gene Expression Omnibus (GEO) database (21) and ENCODE (22) (Supplementary Table S1). Trim Galore (v0.6.6 https://www.bioinformatics.babraham.ac.uk/projects/trim_galore/) was applied to raw data for adaptor trimming. FastQC (23) was used for quality control. All clean data was aligned to the corresponding genomes using Bowtie2 (v2.3.5.1) (24) and SAMtools (25) programs. Bam files were used to call peak by MACS2 (26) with *q* < 0.01. After that, the consensus conserved motifs of Pol III-transcribed ncRNAs were then *de novo* identified by HOMER (27) from peaks.

### Annotation of Pol III-transcribed ncRNAs

Human (hg38) and Mouse (mm10) genome were downloaded from UCSC Genome Brower (28), and their gene annotations were downloaded from GENCODE (29) and UCSC Genome Browser (28). All Pol III-transcribed ncRNAs were extracted and annotated from the RefSeq, GENCODE and RepeatMasker (also called rmsk) annotation data sets. Pol III-transcribed ncRNAs were classified into the following biotypes: 5S rRNA, tRNA, vaultRNA, YRNA scRNA, U6 snRNA, SNAR snRNA, RMRP ribozyme, RPPH1 ribozyme, 7SL srpRNA, BC200 scRNA, 7SK snRNA and Alu for human, as well as 4.5S scRNA, 5S rRNA, 7SK snRNA, 7SL srpRNA, RMRP ribozyme, RPPH1 ribozyme, tRNA, U6 snRNA, vaultRNA, YRNA scRNA and Alu for mouse.

### Association analysis of Pol III-derived ncRNAs and RBPs

RBP binding sites of human and mouse were curated from starBase (30), ENCODE (22) and POSTAR2 (31) (Supplementary Table S2). Peaks came from the same dataset were merged by BEDTools (32) and further applied for strand specific intersecting with Pol III-derived ncRNAs. They were considered as interacting pairs if the overlap length is >20% of the peaks.

### Distribution of RNA modifications and mutation residues on Pol III-derived ncRNAs

RNA modifications of human and mouse were downloaded from RMBase (16) that included 40 RNA modification types (Supplementary Table S2). Cancer-related single-nucleotide variations (SNVs) were curated from COSMIC (33) and published literatures (34,35), that were classified into Substitution (Subs), Deletion (Del), Insertion (Ins), Duplication (Dup), Deletion- Insertion (DelIns) and Inversion (Inv) etc. All above-mentioned sites were intersected with the Pol III-derived ncRNAs with BEDTools.

### Expression profiles of Pol III-transcribed ncRNAs

sRNA-seq datasets of 75 cell lines and tissues were retrieved from ENCODE, and RNA-seq datasets of 28 tumor types were curated from TCGA (19). The featureCounts software (v1.6.0) (36) was applied to bam files for transcript count quantifications. And the raw counts data was recomputed as the Fragments Per Kilobase of transcript per Million mapped reads (FPKM) values to calculate the expression of transcripts. The expression value in different normal tissues or tumors was normalized by *z*-score or mean.

### tsRNAs produced from Pol III-transcribed tRNAs in tumors

tsRNAs were downloaded from tRF2Cancer database (37) and they were classified into six different types including tiRNA-5, tiRNA-3, tRF-5, tRF-3, tRF-i and tRF-1 according to the position of cleavage site. These sites were selected with a significant enrichment of small RNAs with *P*-value < 0.01 (binomial distribution). All these tsRNAs were mapped to the Pol III-derived tRNAs.

### Evolutionary conservation of Pol III-transcribed ncRNAs in different species

All human and mouse Pol III-derived ncRNAs sequences were extracted from the corresponding genome and then were aligned to each other by blastn (v2.12.0) (38). ‘MaxLen’ was defined as the longer length of ncRNA and the reference. Then the aligned pairs whose overlapped length was greater than 90% of maxLen were considered as conserved candidates. Based on that, after filtering with *E*-value <1e–5 and accuracy (exact matches / Identities) > 85%, evolutionary conserved ncRNAs were classified into three types: (i) ‘query-full’ means the whole ncRNA was mapped to the reference; (ii) ‘ref-full’ means part of ncRNA was aligned to the whole reference; (iii) ‘part’ is that ncRNA and the reference are partially matched.

### Database and web interface implementation

All data sets were processed and stored in a MySQL Database Management System. The database query and user interfaces were developed using PHP and JavaScript. The query result tables are based on jQueryUI and DataTables, which is a highly flexible tool for sorting and filtering the search result. The diagrams in the web pages are implemented by Highcharts.

## DATABASE CONTENT AND WEB INTERFACE

### The annotation and identification of regulatory relationships between TF and Pol III-transcribed ncRNAs

To explore the regulatory relationships of between TF and Pol III-transcribed ncRNAs, the gene locus of the ncRNAs were intersected with all Pol III-associated TFs (Supplementary Table S1). In total, we identified 51 097 ncRNAs that regulated by 11 TFs from 10 cells in human (Table 1), and 8234 ncRNAs regulated by four TFs from 4 cells in mouse (Table 2).

**Table 1.** The statistics of Pol III-transcribed ncRNAs mediated by different TFs in human

TFs	BRF1	POLR3D	TFIIIC-220	TBP	BRF2	POLR3GL	BDP1	TFIIIC-110	POLR3G	TFIIIC63	RPC155
Alu	68	28273	37	9864	46	10	378	13070	140	45	4620
Pol3_5S_rRNA	34	34	0	34	0	4	34	34	34	34	34
Pol3_5S_rRNA_pseudogene	12	33	9	109	0	1	23	100	14	12	107
Pol3_5S_rRNA_rmsk	34	64	12	164	0	3	48	149	35	36	165
Pol3_7SK_snRNA	0	2	0	2	2	2	2	0	2	0	2
Pol3_7SK_snRNA_pseudogene	0	12	0	14	1	0	3	0	0	0	29
Pol3_7SK_snRNA_rmsk	0	18	0	19	2	1	4	1	1	0	30
Pol3_7SL_srpRNA	3	4	0	4	0	3	3	4	3	0	4
Pol3_7SL_srpRNA_pseudogene	0	27	0	7	0	1	1	6	2	0	14
Pol3_7SL_srpRNA_rmsk	1	34	0	20	0	1	2	6	2	0	17
Pol3_BC200_scRNA	0	1	0	1	0	0	1	1	0	0	1
Pol3_BC200_scRNA_rmsk	0	6	0	2	0	0	0	4	0	0	3
Pol3_RMRP_ribozyme	0	2	0	2	2	2	2	2	2	0	2
Pol3_RPPH1_ribozyme	0	1	0	1	1	0	1	1	0	0	1
Pol3_SNAR_snRNA	23	16	0	28	0	0	28	26	17	0	24
Pol3_U6_snRNA	0	10	0	10	10	4	10	4	10	0	11
Pol3_U6_snRNA_pseudogene	0	36	0	51	6	0	24	2	2	0	71
Pol3_U6_snRNA_rmsk	0	26	0	25	4	1	9	3	3	0	33
Pol3_YRNA_scRNA	0	4	0	4	4	1	4	1	4	0	4
Pol3_YRNA_scRNA_pseudogene	0	1	0	4	0	0	1	0	0	0	5
Pol3_YRNA_scRNA_rmsk	0	29	0	54	6	1	11	1	4	0	70
Pol3_tRNA	349	464	63	510	4	147	436	468	346	243	480
Pol3_tRNA_rmsk	114	193	15	270	8	43	171	229	119	78	230
Pol3_vaultRNA	4	4	0	4	0	3	4	4	4	0	4
Pol3_vaultRNA_pseudogene	0	0	0	1	0	0	0	0	0	0	1

**Table 2.** The statistics of Pol III-transcribed ncRNAs mediated by different TFs in mouse

TFs	POLR3GL	POLR3D	TBP	POLR3G
Alu	61	616	6768	83
Pol3_4.5S_scRNA	1	1	1	1
Pol3_4.5S_scRNA_rmsk	37	110	177	49
Pol3_5S_rRNA	34	60	80	31
Pol3_5S_rRNA_rmsk	40	80	117	37
Pol3_7SK_snRNA	1	1	1	1
Pol3_7SK_snRNA_rmsk	4	5	13	4
Pol3_7SL_srpRNA	4	5	6	4
Pol3_7SL_srpRNA_rmsk	2	3	8	2
Pol3_RMRP_ribozyme	2	2	2	2
Pol3_RPPH1_ribozyme	1	1	1	1
Pol3_U6_snRNA	5	34	135	5
Pol3_U6_snRNA_pseudogene	1	1	1	1
Pol3_U6_snRNA_rmsk	4	4	33	4
Pol3_YRNA_scRNA	2	2	3	2
Pol3_YRNA_scRNA_rmsk	2	2	7	2
Pol3_tRNA	77	84	103	79
Pol3_tRNA_rmsk	267	312	500	279
Pol3_vaultRNA	1	1	1	1

In TF–RNA browser page, users can explore any items interested them to get the relationships of TF–RNA, such as different Pol III transcription factors and different experiments. For users’ convenience, we also provide a main search box in the right panel to provide a quick search function of ncRNA symbol or other items. Moreover, for each TF interacted Pol III-transcribed ncRNA, we provide an outlink to a new page showing the detail information including genomic loci, gene name, type and the fragments that bound by TF. We also offer one search tool for users in the ‘Tool’ page and they can input gene location, gene sequence or gene name to further investigate whether it hits an annotated Pol III-associated ncRNA in the database. In addition, we provide the consensus sequences of the Pol III-associated TFs in the ‘Motif’ page.

### Exploration of the interactome between Pol III-derived ncRNAs and RBPs

Accumulating evidence demonstrated that RNA–RBP interactions play crucial roles in the RNA metabolism, including transcription, processing, function and degradation (39,40). However, researches on the relationships between Pol III-derived ncRNAs and RBPs lack systematic investigation. Therefore, we intersected tens of millions of RBP binding sites of 283 RBPs for human and mouse with the Pol III-derived ncRNAs and identified over 20 000 interacting pairs covered 102 339 RBP binding sites of 184 RBPs for human, and >10 000 sites of 54 RBPs for mouse. On the ‘RNA-RBP’ page, users can browse the Pol III-derived ncRNA-RBP interactions by ncRNA type. Similarly, this page also provides a quick search module for users to inquire interested ncRNA by its RNA symbol.

### The map of RNA modifications on Pol III-transcribed ncRNAs

RNA modifications widely occur in diverse RNA molecules including Pol III-derived ncRNAs and play vital biological roles. To explore the transcriptome-wide landscape of RNA modifications on Pol III-transcribed ncRNAs, we curated about a million RNA modifications covered 40 types of human and mouse and mapped these sites to Pol III-transcribed ncRNAs. Finally, we characterized the distribution of total 32 types of RNA modifications on these ncRNAs, which contained 7,017 and 2725 RNA modifications of human (Table 3) and mouse (Table 4) respectively. We also found most of RNA modifications, including N1-Methyladenosine (m1A), 5-methylcytosine (m5C), N6-methyladenosine (m6A), 2′-O-Methylation (2′-O-Me), Pseudouridine (Ψ) and A-to-I editing (A-I), tend to decorate tRNA, U6 snRNA, 5S rRNA, 7SL srpRNA and Alu, among that tRNAs were modified by the most abundant and varied RNA modifications, which may trigger a series of downstream biological reactions. Our analysis results will shed the light of the post-transcriptional modification mechanisms of expressed Pol III-transcribed ncRNAs.

**Table 3.** The RNA modification map of Pol III-transcribed ncRNAs of human

modType	ac4C	A-I	Am	Cm	Gm	Um	Y	m1A	m5C	m6A	m7G	others
Alu	0	744	0	1	1	1	2	1	87	209	0	0
Pol3_5S_rRNA	0	0	0	2	0	0	0	0	2	2	0	0
Pol3_5S_rRNA_pseudogene	0	0	0	0	0	0	0	0	24	13	0	0
Pol3_5S_rRNA_rmsk	0	0	0	1	0	0	0	0	31	15	0	0
Pol3_7SK_snRNA	0	0	0	0	0	2	2	0	2	2	0	0
Pol3_7SK_snRNA_pseudogene	0	0	0	0	0	0	0	0	1	8	0	0
Pol3_7SK_snRNA_rmsk	0	0	0	0	0	1	1	0	2	8	0	0
Pol3_7SL_srpRNA	0	2	0	0	0	2	0	0	4	4	0	0
Pol3_7SL_srpRNA_pseudogene	0	30	0	0	0	0	0	1	4	10	0	0
Pol3_7SL_srpRNA_rmsk	0	42	0	1	0	1	0	1	6	13	0	0
Pol3_BC200_scRNA	0	0	0	0	0	0	0	0	1	1	0	0
Pol3_BC200_scRNA_rmsk	0	6	0	0	0	0	0	0	0	0	0	0
Pol3_RMRP_ribozyme	0	0	2	0	0	0	0	0	0	2	0	0
Pol3_RPPH1_ribozyme	0	0	0	0	0	0	0	0	1	1	0	0
Pol3_SNAR_snRNA	0	0	0	0	0	0	0	0	4	15	0	0
Pol3_U6_snRNA	0	0	10	10	9	9	11	0	0	0	0	0
Pol3_U6_snRNA_pseudogene	0	1	189	79	155	39	87	0	0	6	0	0
Pol3_U6_snRNA_rmsk	0	0	120	38	95	21	55	0	0	3	0	0
Pol3_YRNA_scRNA	0	0	0	0	0	1	0	0	2	3	0	0
Pol3_YRNA_scRNA_pseudogene	0	0	0	0	0	0	0	0	0	2	0	0
Pol3_YRNA_scRNA_rmsk	0	0	0	0	0	1	0	0	1	9	0	0
Pol3_tRNA	17	15	1	42	49	29	258	329	288	19	77	524
Pol3_tRNA_rmsk	8	4	0	17	11	12	127	124	89	11	26	269
Pol3_vaultRNA	0	0	0	0	0	0	0	0	3	2	0	0

**Table 4.** The RNA modification map of Pol III-transcribed ncRNAs of mouse

modType	A-I	Am	Cm	Gm	Um	Y	m1A	m5C	m6A	m7G	others
Alu	17	0	0	0	0	0	0	5	41	0	0
Pol3_4.5S_scRNA_rmsk	1	0	0	0	0	0	0	0	1	0	0
Pol3_5S_rRNA	2	0	0	0	0	0	0	0	21	0	0
Pol3_5S_rRNA_rmsk	2	0	0	0	0	0	0	0	22	0	0
Pol3_7SK_snRNA	0	0	0	0	0	0	0	0	1	0	0
Pol3_7SK_snRNA_rmsk	0	0	0	0	0	0	0	0	7	0	0
Pol3_7SL_srpRNA	0	0	0	0	0	0	0	2	3	0	0
Pol3_7SL_srpRNA_rmsk	0	0	0	0	0	0	0	0	1	0	0
Pol3_RMRP_ribozyme	0	0	0	0	0	0	0	0	2	0	0
Pol3_RPPH1_ribozyme	0	0	0	0	0	0	0	1	1	0	0
Pol3_U6_snRNA	0	125	209	0	0	238	0	0	23	0	362
Pol3_U6_snRNA_rmsk	0	25	58	0	0	66	0	0	6	0	88
Pol3_tRNA	1	0	8	5	4	19	18	20	0	15	100
Pol3_tRNA_rmsk	16	0	47	25	7	139	82	93	13	53	543
Pol3_vaultRNA	0	0	0	0	0	0	0	0	1	0	0

### Decoding the tsRNAs derived from Pol III-transcribed tRNAs in diverse tumor types

tRNA-derived small RNAs (tsRNAs), which participate in various physiological and pathological processes and function as key players in the occurrence and development of tumors, are generated from mature or precursor Pol III-transcribed tRNAs. (41). To help users better investigate the biological roles of Pol III-transcribed tRNAs, we curated about 5000 tsRNAs in 32 tumor types. Finally, we obtained 4336 Pol III tRNA-derived tsRNAs and provided them with a series of information, including the corresponding tsRNA ID, tumor specificity, genomic coordinate, type and sequence.

### Exploring the expression profiles of Pol III-transcribed ncRNAs in diverse normal tissues and tumor types

Specific quantitative expression of ncRNAs in certain tissues or cells is often used to study the function of ncRNAs in biological processes. To do this for Pol III-transcribed ncRNAs, we provided two webpages to quantitate their expression in normal cells or tissues and tumors. Two normalized expression values, including mean value and z-score value, of these ncRNAs were available for understanding the relative expression in different tissues or tumor types. Firstly, we provided ‘Expression’ page to explore the expression profiles of these ncRNAs in 75 cells and normal tissues and the corresponding detail page to display the real expression value of ncRNAs and their variance. In addition, we provided ‘Pan-Cancer’ page including two sub-pages: ‘ncRNA case’ sub-page is used to investigate the expression profiles of these ncRNAs in 28 tumor types and their adjacent tissues; and ‘cancer case’ sub-page shows the expression profile of the certain ncRNA in all samples of a specific tumor type.

### Mutation map of Pol III-transcribed ncRNAs in various diseases

We further elucidated the map of mutation residues on these ncRNAs in different cancers and diseases. We collected 6 mutation types with about 10 million residues and mapped them to Pol III-transcribed ncRNAs. Finally, we obtained 10 529 mutation residues on 10 610 ncRNAs, including 14 552 Subs, 329 Ins, 605 Del and 3 DelIns.

### Evolutionarily conservation of Pol III-transcribed ncRNAs in mammals

Previous studies suggested that the common features of Pol III-transcribed ncRNAs affect their structure and downstream functions, among which the role in the assembly and function of RNPs is evolutionarily conserved (42,43). In addition, we deciphered interspecies evolutionarily conservation of these ncRNAs by aligning ncRNAs to each other's transcriptome. After filtering with *E*-value < 1e–5 and accuracy >85%, we finally identified 2528 and 1402 evolutionary conserved ncRNAs for human and mouse respectively, which were classified into three types: ‘query-full’, ‘ref-full’ and ‘part’.

## DISCUSSION AND CONCLUSIONS

By integrating a large set of ChIP-seq, CLIP-seq, sRNA-seq, epitranscriptome data, functional annotations and public resources, Pol3Base provided the most comprehensive transcription, expression, epitranscriptome, interaction and mutation profiles of Pol III ncRNAs in human and mouse. Currently, only tRNA databases (e.g. GtRNAdb (44), tRNAdb (45), T-psi-C (46), tRNADB-CE (47)) contain one type of ncRNA (tRNA) transcribed by Pol III (Supplementary Table S3), other ncRNA databases such as miRBase (48), circBase (49) and NONCODE (50) focus on other specific type of ncRNAs. Compare with the above-mentioned databases (Supplementary Table S3), Pol3Base for the first time to systematically integrate all types of Pol III-transcribed ncRNAs, includes tRNAs, 5S RNAs, 7SL RNAs, 7SK RNAs, Alu RNA, U6 RNAs, Y RNAs, RMRP, RPPH1, BC200, SNAR, vaultRNA, etc. Importantly, we construct pol3Base to associate these Pol III-transcribed ncRNAs with various scientific frontiers, such as epitranscriptome, interactome, Pan-Cancer, disease and tsRNAs.

Pol3Base is the first database for decoding the interactome, expression, evolution, epitranscriptome and disease variations of Pol III-transcribed ncRNAs. The advances of our Pol3Base database are as follows. (a) Pol3Base is the first database providing the most comprehensive transcriptional regulatory networks of Pol III-transcribed ncRNAs by analyzing transcription factor binding maps identified from high-throughput ChIP-seq datasets. (b) We integrated and analyzed 240 CLIP-seq datasets to explore the binding pattern of RBPs on Pol III-transcribed ncRNAs. It may help biologists to investigate the function of Pol III-transcribed ncRNAs. (c) We constructed ‘modification’ module to provide a quick overview of characterization of RNA modifications on the Pol III-transcribed ncRNAs by analyzing epitranscriptome sequencing data. These results will help to reveal the post-transcriptional regulation of Pol III-transcribed ncRNAs caused by RNA modifications. (d) We investigated the expression of Pol III-transcribed ncRNAs across cancer tissues and cell lines. We also performed pan-cancer analysis on Pol III-transcribed ncRNAs based on expression data from TCGA small RNA-seq experiments of 28 different tumor types and normal tissues. This will generate numerous differentially expressed ncRNAs or cell/tissue-specific ncRNAs for the functional study of bench biologists. (e) Pol3Base also for the first time to systematically characterize somatic mutations on Pol III-transcribed ncRNAs. This will help researcher to discover disease-related ncRNAs for further functional validation. (f) We built ‘Evolution’ module to illustrate evolutionary conservation of Pol III-transcribed ncRNAs in different mammals to facilitate function exploration of these ncRNAs. (g) Pol3Base provides a variety of interfaces and graphic visualizations to facilitate analysis and exploration of functions and mechanisms of Pol III-transcribed ncRNAs. In all, Pol3Base provides considerable richness and enormous convenience and will become increasingly important for the study of Pol III transcriptome.

## FUTURE DIRECTIONS

Pol3Base will continue to improve the computer server performance and built an automatic pipeline for storing and analyzing new high-throughput data generated from CLIP-seq, ChIP-seq, sRNA-seq and Ribo-seq technologies, and then to decipher biological function and mechanism, regulatory networks and translational potential of Pol III-transcribed ncRNAs. We will also develop new tools to integrate more annotation data, high-throughput sequencing data and additional species to further expand this resource. We will continually maintain and update the resource every 3 months or whenever new data sets are released in public databases.

## DATA AVAILABILITY

Pol3Base is freely available at http://rna.sysu.edu.cn/pol3base/ or http://biomed.nscc-gz.cn/DB/Pol3Base/. The Pol3Base data files can be downloaded and used in accordance with the GNU Public License and the license of primary data sources.

## Supplementary Material

gkab1033_Supplemental_File
