# Supplementary material for: Pol3Base: a resource for decoding the interactome, expression, evolution, epitranscriptome and disease variations of Pol III-transcribed ncRNAs
Source: Nucleic Acids Res. 2021 Nov 8;50(D1):D279–86. doi: 10.1093/nar/gkab1033 (PMC8728242; doi:10.1093/nar/gkab1033)
Supplement: gkab1033_Supplemental_File [file gkab1033_supplemental_file.pdf]

Supplementary Data

Pol3Base: A Resource for Decoding the Interactome, Expression, Evolution, Epitranscriptome and Disease Variations of Pol III-transcribed ncRNAs

Authors:

Li Cai[1,*], Jiajia Xuan[1,*], Qiao Lin[1], Junhao Wang[1], Shurong Liu[1], Fangzhou Xie[1], Lingling Zheng[1,*], Bin Li[1,*], Lianghu Qu[1,*], Jianhua Yang[1,2*]

Supplementary Table 1. The publicly available Pol III-associated ChIP-seq datasets collected in Pol3Base.

Species	TF	Sample	Datasets	References
H. sapiens	BDP1	IMR90	GSM1160643	(1)
		IMR90hTert	GSM454596	(2)
		HeLa-S3	GSM935486	(3)
		K562	GSM935594	(3)
	BRF1	IMR90hTert	GSM454597	(2)
		HeLa	GSM501711	(4)
		HeLa-S3	GSM509049	(5)
		HeLa-S3	GSM935582	(3)
		K562	GSM935595	(3)
	BRF2	HeLa-S3	GSM509050	(5)
		HeLa-S3	GSM935435	(3)
		K562	GSM935490	(3)
	POLR3D	IMR90	GSM1160644	(1)
		IMR90hTert	GSM1906346	(6)
		IMR90hTert	GSM1906347	(6)
		IMR90hTert	GSM1906349	(6)
		IMR90hTert	GSM1906350	(6)
		IMR90hTert	GSM1906351	(6)
		IMR90hTert	GSM1906352	(6)
		IMR90hTert	GSM1906361	(6)
		IMR90hTert	GSM1906362	(6)
		IMR90hTert	GSM1906363	(6)

		IMR90hTert	GSM454595	(2)
	POLR3G	IMR90	GSM1160645	(1)
		K562	GSM487428	(7)
		GM12878	GSM487429	(7)
		HeLa-S3	GSM509052	(5)
		HeLa-S3	GSM509053	(5)
		Jurkat-E6.1	GSM509057	(5)
	POLR3GL	IMR90	GSM1160646	(1)
	RPC155	HeLa	GSM501710	(4)
		CD4+Tcells	GSM501714	(4)
		HeLa-S3	GSM935489	(3)
	TBP	GM12878	GSM935277	(3)
		HepG2	GSM935280	(3)
		H1-hESC	GSM935303	(3)
		K562	GSM935495	(3)
		HeLa-S3	GSM935606	(3)
	TFIIIC-110	HeLa-S3	GSM935342	(3)
		K562	GSM935343	(3)
	TFIIIC-220	HeLa	GSM501712	(4)
	TFIIIC63	HeLa-S3	GSM509056	(5)
M. musculus	POLR3D	C57/BL6	GSM1160649	(1)
		C57/BL6	GSM1160650	(1)
		Hepa1-6	GSM1160656	(1)
		Hepa1-6	GSM1160657	(1)
	POLR3G	C57/BL6	GSM1160651	(1)
		C57/BL6	GSM1160652	(1)
		Hepa1-6	GSM1160658	(1)
		Hepa1-6	GSM1160659	(1)
	POLR3GL	C57/BL6	GSM1160653	(1)
		C57/BL6	GSM1160654	(1)
		Hepa1-6	GSM1160660	(1)
		Hepa1-6	GSM1160661	(1)
	TBP	CH12	GSM912900	(3)

	MEL	GSM912913	(3)

Supplementary Table 2. The publicly available datasets collected in Pol3Base.

Data Type	Resource	References
RNA-RBP	starBase v2.0, ENCODE, POSTAR2	(3) (8) (9)
RNA-Mod	RMBase v2.0	(10)
Expression	ENCODE, TCGA	(3) (11)
Pan-Cancer	TCGA	(11)
Disease	COSMIC	(12-14)
tsRNA	tRF2Cancer	(15)

Supplementary Table 3. The differences between Pol3Base and other published ncRNA databases.

Features	Pol3Base	GtRNAdb	tRNAdb	T-psi-C	tRNADB-CE
ncRNA types	12 types of ncRNAs	tRNA	tRNA	tRNA	tRNA
RBP	Yes	No	No	No	No
Modification	Yes	No	No	No	No
Expression	Yes	No	No	No	No
Cancer	Yes	No	No	No	No
Disease	Yes	No	No	No	No
Evolution	Yes	Yes	Yes	Yes	Yes
Motif	Yes	No	No	No	No

References:

1. Renaud, M., Praz, V., Vieu, E., Florens, L., Washburn, M.P., l'Hote, P. and Hernandez, N. (2014) Gene duplication and neofunctionalization: POLR3G and POLR3GL. *Genome research*, **24**, 37-51.
2. Canella, D., Praz, V., Reina, J.H., Cousin, P. and Hernandez, N. (2010) Defining the RNA polymerase III transcriptome: Genome-wide localization of the RNA polymerase III transcription machinery in human cells. *Genome Res*, **20**, 710-721.
3. Davis, C.A., Hitz, B.C., Sloan, C.A., Chan, E.T., Davidson, J.M., Gabdank, I., Hilton, J.A., Jain, K., Baymuradov, U.K., Narayanan, A.K. *et al.* (2018) The Encyclopedia of DNA elements

(ENCODE): data portal update. *Nucleic Acids Res*, **46**, D794-D801.

4. Barski, A., Chepelev, I., Liko, D., Cuddapah, S., Fleming, A.B., Birch, J., Cui, K., White, R.J. and Zhao, K. (2010) Pol II and its associated epigenetic marks are present at Pol III-transcribed noncoding RNA genes. *Nat Struct Mol Biol*, **17**, 629-634.
5. Oler, A.J., Alla, R.K., Roberts, D.N., Wong, A., Hollenhorst, P.C., Chandler, K.J., Cassiday, P.A., Nelson, C.A., Hagedorn, C.H., Graves, B.J. *et al.* (2010) Human RNA polymerase III transcriptomes and relationships to Pol II promoter chromatin and enhancer-binding factors. *Nat Struct Mol Biol*, **17**, 620-628.
6. Orioli, A., Praz, V., Lhote, P. and Hernandez, N. (2016) Human MAF1 targets and represses active RNA polymerase III genes by preventing recruitment rather than inducing long-term transcriptional arrest. *Genome research*, **26**, 624-635.
7. Raha, D., Wang, Z., Moqtaderi, Z., Wu, L., Zhong, G., Gerstein, M., Struhl, K. and Snyder, M. (2010) Close association of RNA polymerase II and many transcription factors with Pol III genes. *Proc Natl Acad Sci U S A*, **107**, 3639-3644.
8. Li, J.H., Liu, S., Zhou, H., Qu, L.H. and Yang, J.H. (2014) starBase v2.0: decoding miRNA-ceRNA, miRNA-ncRNA and protein-RNA interaction networks from large-scale CLIP-Seq data. *Nucleic Acids Res*, **42**, D92-97.
9. Zhu, Y., Xu, G., Yang, Y.T., Xu, Z., Chen, X., Shi, B., Xie, D., Lu, Z.J. and Wang, P. (2019) POSTAR2: deciphering the post-transcriptional regulatory logics. *Nucleic Acids Res*, **47**, D203-D211.
10. Xuan, J.J., Sun, W.J., Lin, P.H., Zhou, K.R., Liu, S., Zheng, L.L., Qu, L.H. and Yang, J.H. (2018) RMBase v2.0: deciphering the map of RNA modifications from epitranscriptome sequencing data. *Nucleic Acids Res*, **46**, D327-D334.
11. Weinstein, J.N., Collisson, E.A., Mills, G.B., Shaw, K.R.M., Ozenberger, B.A., Ellrott, K., Shmulevich, I., Sander, C., Stuart, J.M. and Network, C.G.A.R. (2013) The cancer genome atlas pan-cancer analysis project. *Nature genetics*, **45**, 1113.
12. Tate, J.G., Bamford, S., Jubb, H.C., Sondka, Z., Beare, D.M., Bindal, N., Boutselakis, H., Cole, C.G., Creatore, C., Dawson, E. *et al.* (2019) COSMIC: the Catalogue Of Somatic Mutations In Cancer. *Nucleic Acids Res*, **47**, D941-D947.
13. Alexandrov, L.B., Nik-Zainal, S., Wedge, D.C., Aparicio, S.A., Behjati, S., Biankin, A.V., Bignell, G.R., Bolli, N., Borg, A., Borresen-Dale, A.L. *et al.* (2013) Signatures of mutational processes in human cancer. *Nature*, **500**, 415-421.
14. Lawrence, M.S., Stojanov, P., Mermel, C.H., Robinson, J.T., Garraway, L.A., Golub, T.R., Meyerson, M., Gabriel, S.B., Lander, E.S. and Getz, G. (2014) Discovery and saturation analysis of cancer genes across 21 tumour types. *Nature*, **505**, 495-501.
15. Zheng, L.L., Xu, W.L., Liu, S., Sun, W.J., Li, J.H., Wu, J., Yang, J.H. and Qu, L.H. (2016) tRF2Cancer: A web server to detect tRNA-derived small RNA fragments (tRFs) and their expression in multiple cancers. *Nucleic Acids Res*, **44**, W185-193.
